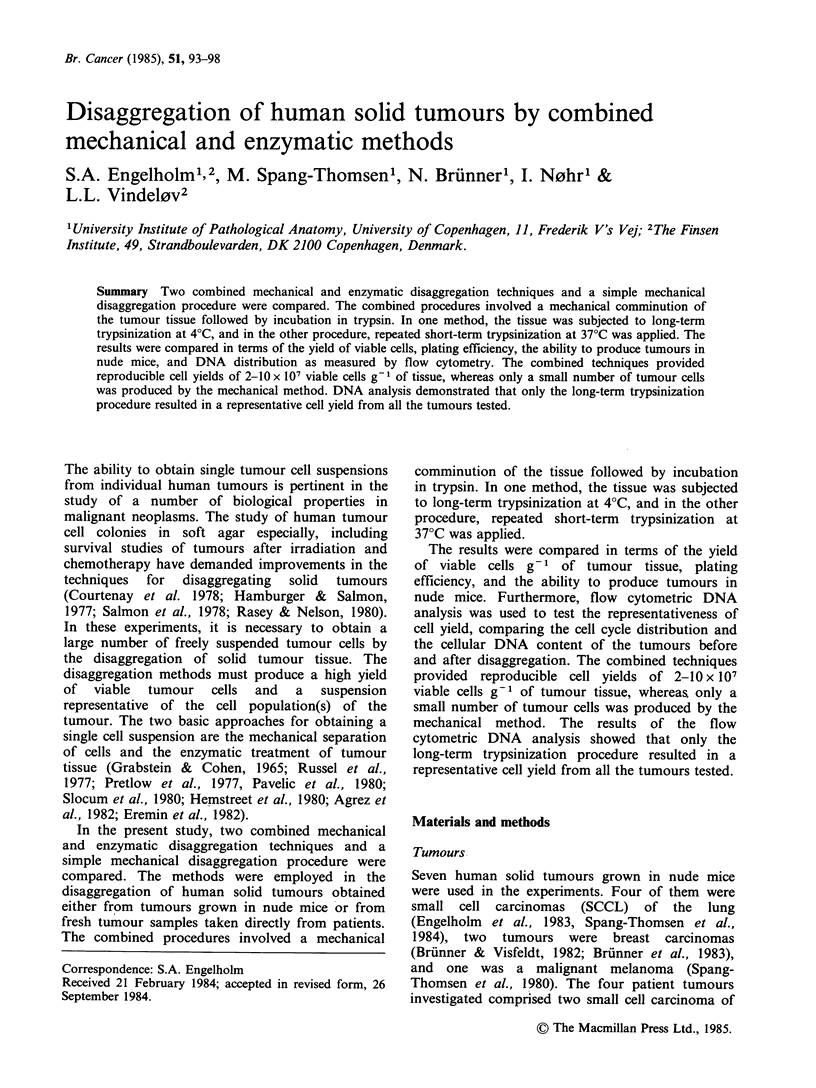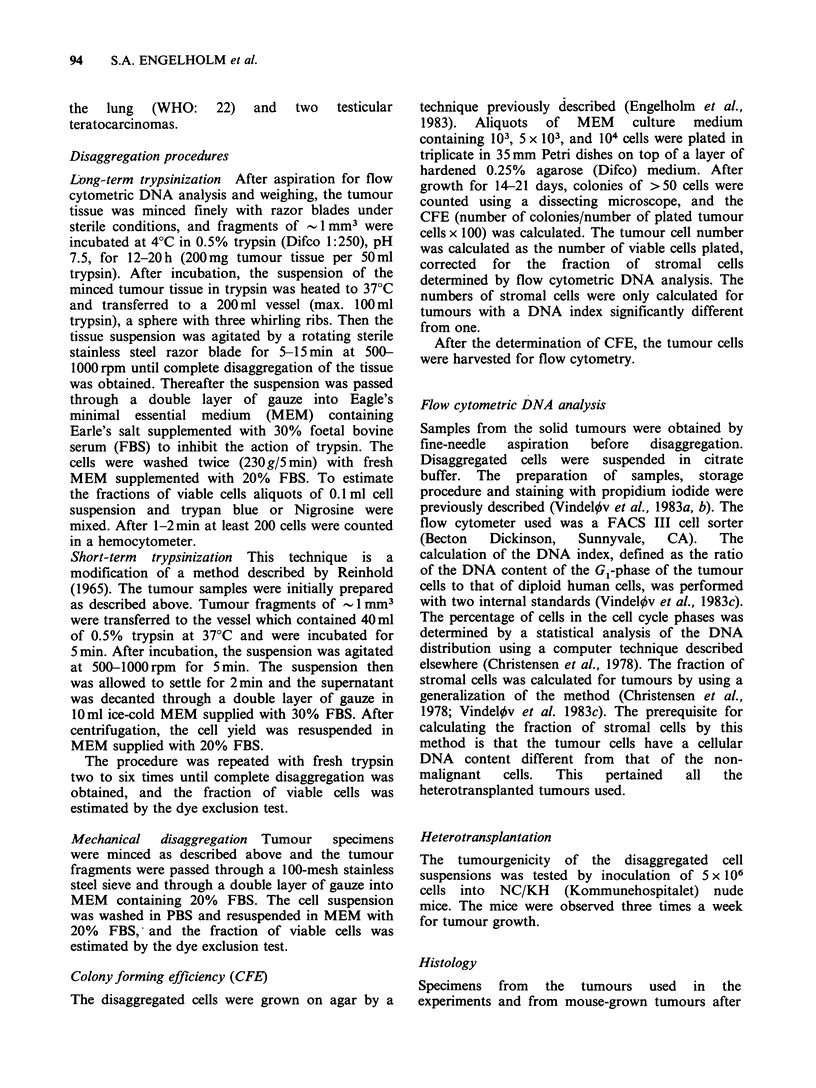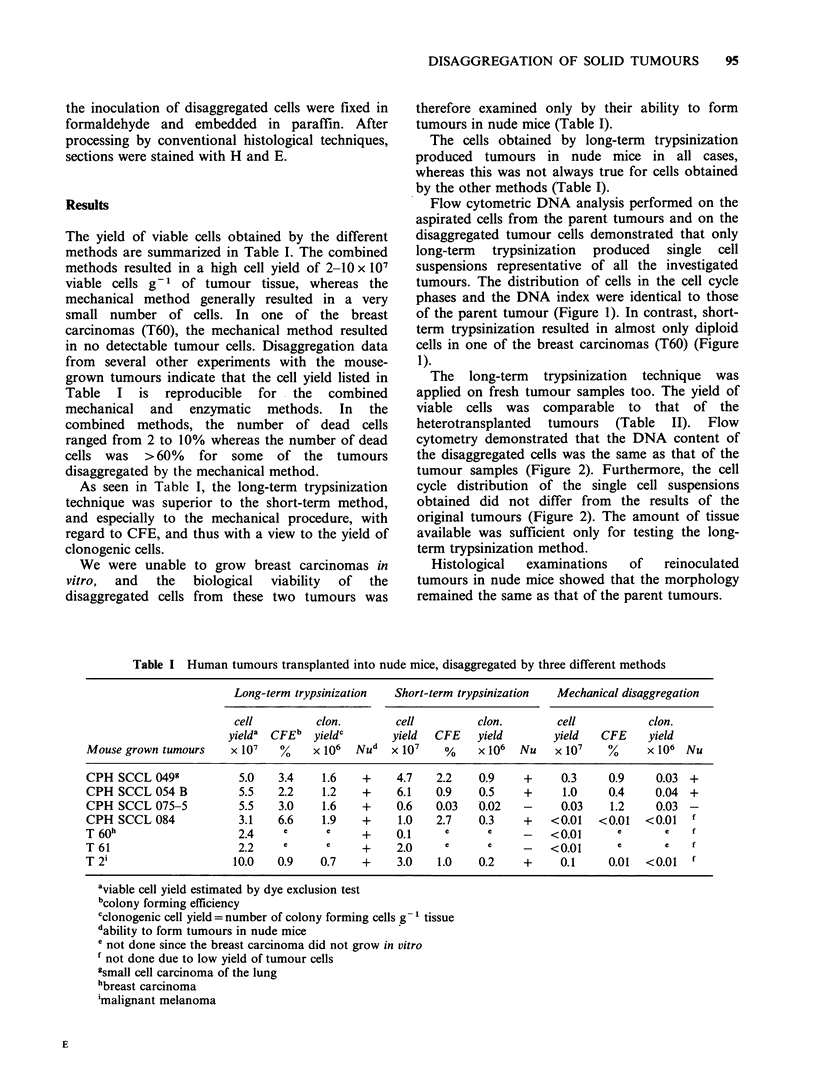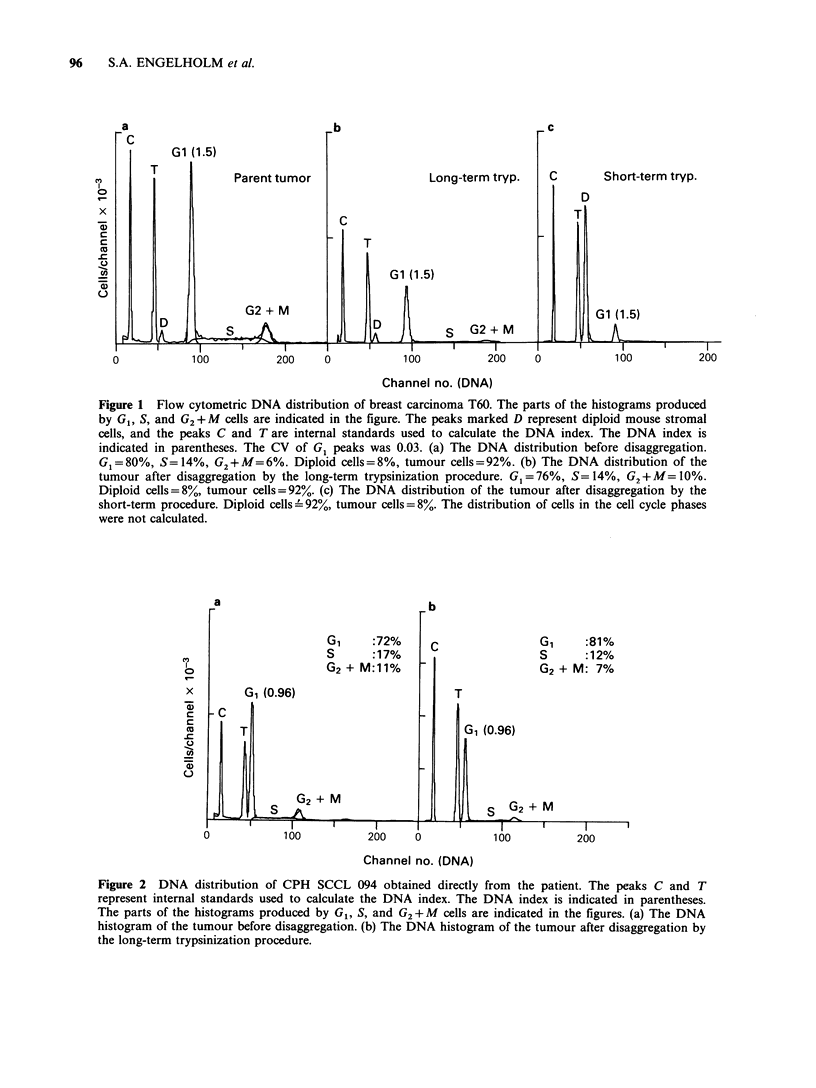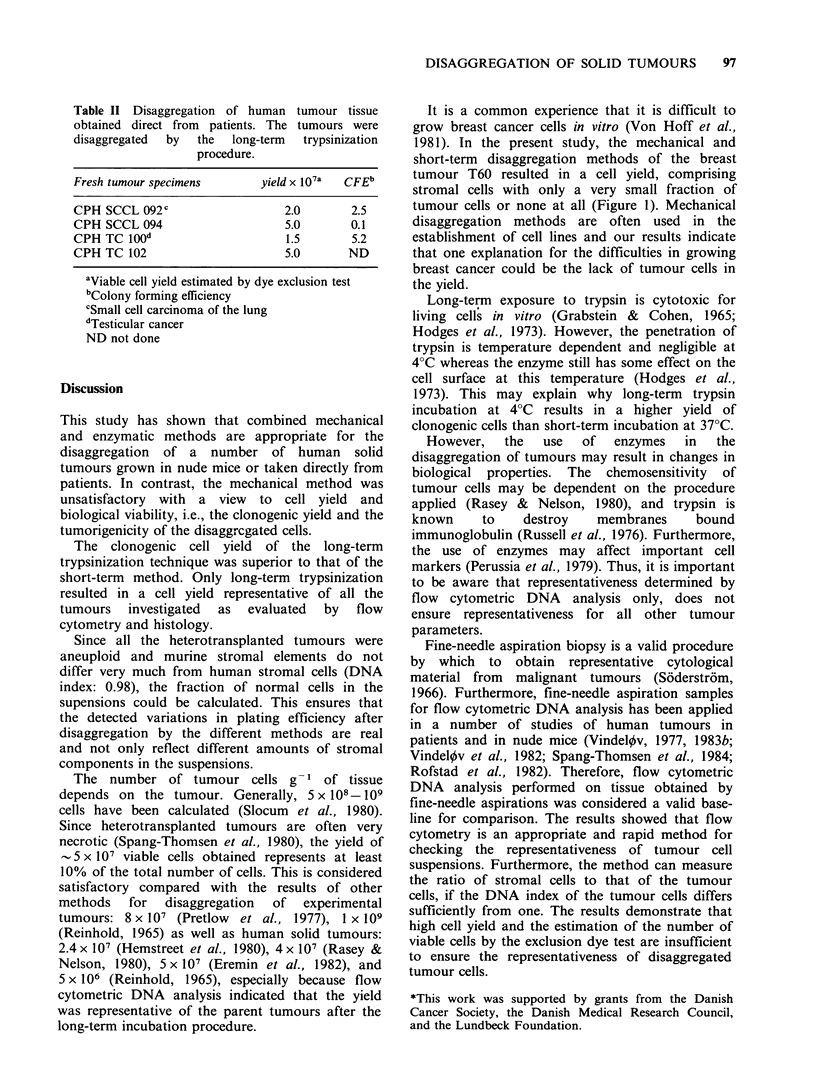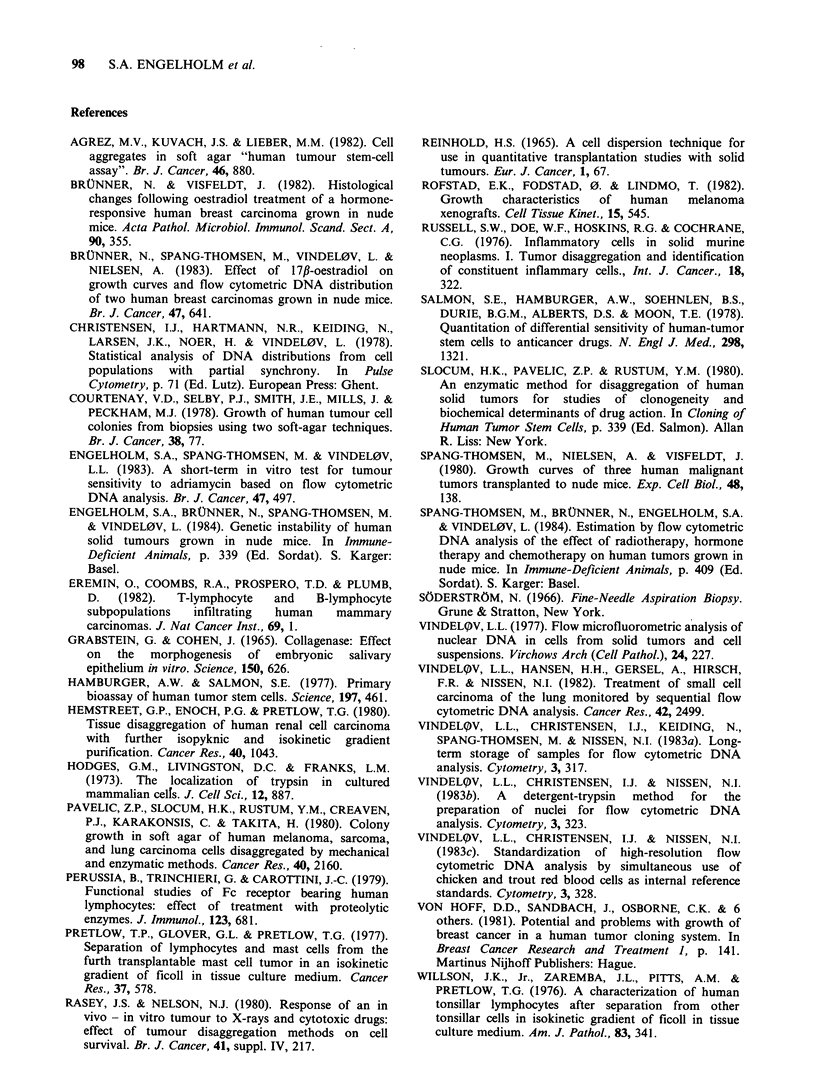# Disaggregation of human solid tumours by combined mechanical and enzymatic methods.

**DOI:** 10.1038/bjc.1985.13

**Published:** 1985-01

**Authors:** S. A. Engelholm, M. Spang-Thomsen, N. Brünner, I. Nøhr, L. L. Vindeløv

## Abstract

Two combined mechanical and enzymatic disaggregation techniques and a simple mechanical disaggregation procedure were compared. The combined procedures involved a mechanical comminution of the tumour tissue followed by incubation in trypsin. In one method, the tissue was subjected to long-term trypsinization at 4 degrees C, and in the other procedure, repeated short-term trypsinization at 37 degrees C was applied. The results were compared in terms of the yield of viable cells, plating efficiency, the ability to produce tumours in nude mice, and DNA distribution as measured by flow cytometry. The combined techniques provided reproducible cell yields of 2-10 X 10(7) viable cells g-1 of tissue, whereas only a small number of tumour cells was produced by the mechanical method. DNA analysis demonstrated that only the long-term trypsinization procedure resulted in a representative cell yield from all the tumours tested.


*Br. Cancer* (1985), **51**, 93–98

### Disaggregation of human solid tumours by combined mechanical and enzymatic methods

S.A. Engelholm[1,2], M. Spang-Thomsen[1], N. Brünner[1], I. Nøhr[1] & L.L. Vindeløv[2]

[1]*University Institute of Pathological Anatomy, University of Copenhagen, 11, Frederik V's Vej;* [2]*The Finsen Institute, 49, Strandboulevarden, DK 2100 Copenhagen, Denmark.*

**Summary** Two combined mechanical and enzymatic disaggregation techniques and a simple mechanical disaggregation procedure were compared. The combined procedures involved a mechanical comminution of the tumour tissue followed by incubation in trypsin. In one method, the tissue was subjected to long-term trypsinization at 4°C, and in the other procedure, repeated short-term trypsinization at 37°C was applied. The results were compared in terms of the yield of viable cells, plating efficiency, the ability to produce tumours in nude mice, and DNA distribution as measured by flow cytometry. The combined techniques provided reproducible cell yields of $2–10 \times 10^7$ viable cells $g^{-1}$ of tissue, whereas only a small number of tumour cells was produced by the mechanical method. DNA analysis demonstrated that only the long-term trypsinization procedure resulted in a representative cell yield from all the tumours tested.

The ability to obtain single tumour cell suspensions from individual human tumours is pertinent in the study of a number of biological properties in malignant neoplasms. The study of human tumour cell colonies in soft agar especially, including survival studies of tumours after irradiation and chemotherapy have demanded improvements in the techniques for disaggregating solid tumours (Courtenay *et al.* 1978; Hamburger & Salmon, 1977; Salmon *et al.*, 1978; Rasey & Nelson, 1980). In these experiments, it is necessary to obtain a large number of freely suspended tumour cells by the disaggregation of solid tumour tissue. The disaggregation methods must produce a high yield of viable tumour cells and a suspension representative of the cell population(s) of the tumour. The two basic approaches for obtaining a single cell suspension are the mechanical separation of cells and the enzymatic treatment of tumour tissue (Grabstein & Cohen, 1965; Russel *et al.*, 1977; Pretlow *et al.*, 1977, Pavelic *et al.*, 1980; Slocum *et al.*, 1980; Hemstreet *et al.*, 1980; Agrez *et al.*, 1982; Eremin *et al.*, 1982).

In the present study, two combined mechanical and enzymatic disaggregation techniques and a simple mechanical disaggregation procedure were compared. The methods were employed in the disaggregation of human solid tumours obtained either from tumours grown in nude mice or from fresh tumour samples taken directly from patients. The combined procedures involved a mechanical comminution of the tissue followed by incubation in trypsin. In one method, the tissue was subjected to long-term trypsinization at 4°C, and in the other procedure, repeated short-term trypsinization at 37°C was applied.

The results were compared in terms of the yield of viable cells $g^{-1}$ of tumour tissue, plating efficiency, and the ability to produce tumours in nude mice. Furthermore, flow cytometric DNA analysis was used to test the representativeness of cell yield, comparing the cell cycle distribution and the cellular DNA content of the tumours before and after disaggregation. The combined techniques provided reproducible cell yields of $2–10 \times 10^7$ viable cells $g^{-1}$ of tumour tissue, whereas only a small number of tumour cells was produced by the mechanical method. The results of the flow cytometric DNA analysis showed that only the long-term trypsinization procedure resulted in a representative cell yield from all the tumours tested.

### Materials and methods

### *Tumours*

Seven human solid tumours grown in nude mice were used in the experiments. Four of them were small cell carcinomas (SCCL) of the lung (Engelholm *et al.*, 1983, Spang-Thomsen *et al.*, 1984), two tumours were breast carcinomas (Brünner & Visfeldt, 1982; Brünner *et al.*, 1983), and one was a malignant melanoma (Spang-Thomsen *et al.*, 1980). The four patient tumours investigated comprised two small cell carcinoma of

Correspondence: S.A. Engelholm
Received 21 February 1984; accepted in revised form, 26 September 1984.

© The Macmillan Press Ltd., 1985.

the lung (WHO: 22) and two testicular teratocarcinomas.

*Disaggregation procedures*

*Long-term trypsinization* After aspiration for flow cytometric DNA analysis and weighing, the tumour tissue was minced finely with razor blades under sterile conditions, and fragments of ~1 mm³ were incubated at 4°C in 0.5% trypsin (Difco 1:250), pH 7.5, for 12–20 h (200 mg tumour tissue per 50 ml trypsin). After incubation, the suspension of the minced tumour tissue in trypsin was heated to 37°C and transferred to a 200 ml vessel (max. 100 ml trypsin), a sphere with three whirling ribs. Then the tissue suspension was agitated by a rotating sterile stainless steel razor blade for 5–15 min at 500–1000 rpm until complete disaggregation of the tissue was obtained. Thereafter the suspension was passed through a double layer of gauze into Eagle's minimal essential medium (MEM) containing Earle's salt supplemented with 30% foetal bovine serum (FBS) to inhibit the action of trypsin. The cells were washed twice (230 *g*/5 min) with fresh MEM supplemented with 20% FBS. To estimate the fractions of viable cells aliquots of 0.1 ml cell suspension and trypan blue or Nigrosine were mixed. After 1–2 min at least 200 cells were counted in a hemocytometer.

*Short-term trypsinization* This technique is a modification of a method described by Reinhold (1965). The tumour samples were initially prepared as described above. Tumour fragments of ~1 mm³ were transferred to the vessel which contained 40 ml of 0.5% trypsin at 37°C and were incubated for 5 min. After incubation, the suspension was agitated at 500–1000 rpm for 5 min. The suspension then was allowed to settle for 2 min and the supernatant was decanted through a double layer of gauze in 10 ml ice-cold MEM supplied with 30% FBS. After centrifugation, the cell yield was resuspended in MEM supplied with 20% FBS.

The procedure was repeated with fresh trypsin two to six times until complete disaggregation was obtained, and the fraction of viable cells was estimated by the dye exclusion test.

*Mechanical disaggregation* Tumour specimens were minced as described above and the tumour fragments were passed through a 100-mesh stainless steel sieve and through a double layer of gauze into MEM containing 20% FBS. The cell suspension was washed in PBS and resuspended in MEM with 20% FBS, and the fraction of viable cells was estimated by the dye exclusion test.

*Colony forming efficiency (CFE)*

The disaggregated cells were grown on agar by a technique previously described (Engelholm *et al.*, 1983). Aliquots of MEM culture medium containing $10^3$, $5 \times 10^3$, and $10^4$ cells were plated in triplicate in 35 mm Petri dishes on top of a layer of hardened 0.25% agarose (Difco) medium. After growth for 14–21 days, colonies of >50 cells were counted using a dissecting microscope, and the CFE (number of colonies/number of plated tumour cells × 100) was calculated. The tumour cell number was calculated as the number of viable cells plated, corrected for the fraction of stromal cells determined by flow cytometric DNA analysis. The numbers of stromal cells were only calculated for tumours with a DNA index significantly different from one.

After the determination of CFE, the tumour cells were harvested for flow cytometry.

*Flow cytometric DNA analysis*

Samples from the solid tumours were obtained by fine-needle aspiration before disaggregation. Disaggregated cells were suspended in citrate buffer. The preparation of samples, storage procedure and staining with propidium iodide were previously described (Vindeløv *et al.*, 1983*a, b*). The flow cytometer used was a FACS III cell sorter (Becton Dickinson, Sunnyvale, CA). The calculation of the DNA index, defined as the ratio of the DNA content of the $G_1$-phase of the tumour cells to that of diploid human cells, was performed with two internal standards (Vindeløv *et al.*, 1983*c*). The percentage of cells in the cell cycle phases was determined by a statistical analysis of the DNA distribution using a computer technique described elsewhere (Christensen *et al.*, 1978). The fraction of stromal cells was calculated for tumours by using a generalization of the method (Christensen *et al.*, 1978; Vindeløv *et al.* 1983*c*). The prerequisite for calculating the fraction of stromal cells by this method is that the tumour cells have a cellular DNA content different from that of the non-malignant cells. This pertained all the heterotransplanted tumours used.

*Heterotransplantation*

The tumourgenicity of the disaggregated cell suspensions was tested by inoculation of $5 \times 10^6$ cells into NC/KH (Kommunehospitalet) nude mice. The mice were observed three times a week for tumour growth.

*Histology*

Specimens from the tumours used in the experiments and from mouse-grown tumours after the inoculation of disaggregated cells were fixed in formaldehyde and embedded in paraffin. After processing by conventional histological techniques, sections were stained with H and E.

### Results

The yield of viable cells obtained by the different methods are summarized in Table I. The combined methods resulted in a high cell yield of $2–10 \times 10^7$ viable cells $g^{-1}$ of tumour tissue, whereas the mechanical method generally resulted in a very small number of cells. In one of the breast carcinomas (T60), the mechanical method resulted in no detectable tumour cells. Disaggregation data from several other experiments with the mouse-grown tumours indicate that the cell yield listed in Table I is reproducible for the combined mechanical and enzymatic methods. In the combined methods, the number of dead cells ranged from 2 to 10% whereas the number of dead cells was >60% for some of the tumours disaggregated by the mechanical method.

As seen in Table I, the long-term trypsinization technique was superior to the short-term method, and especially to the mechanical procedure, with regard to CFE, and thus with a view to the yield of clonogenic cells.

We were unable to grow breast carcinomas *in vitro,* and the biological viability of the disaggregated cells from these two tumours was therefore examined only by their ability to form tumours in nude mice (Table I).

The cells obtained by long-term trypsinization produced tumours in nude mice in all cases, whereas this was not always true for cells obtained by the other methods (Table I).

Flow cytometric DNA analysis performed on the aspirated cells from the parent tumours and on the disaggregated tumour cells demonstrated that only long-term trypsinization produced single cell suspensions representative of all the investigated tumours. The distribution of cells in the cell cycle phases and the DNA index were identical to those of the parent tumour (Figure 1). In contrast, short-term trypsinization resulted in almost only diploid cells in one of the breast carcinomas (T60) (Figure 1).

The long-term trypsinization technique was applied on fresh tumour samples too. The yield of viable cells was comparable to that of the heterotransplanted tumours (Table II). Flow cytometry demonstrated that the DNA content of the disaggregated cells was the same as that of the tumour samples (Figure 2). Furthermore, the cell cycle distribution of the single cell suspensions obtained did not differ from the results of the original tumours (Figure 2). The amount of tissue available was sufficient only for testing the long-term trypsinization method.

Histological examinations of reinoculated tumours in nude mice showed that the morphology remained the same as that of the parent tumours.

**Table I** Human tumours transplanted into nude mice, disaggregated by three different methods

| | *Long-term trypsinization* | | | | *Short-term trypsinization* | | | | *Mechanical disaggregation* | | | |
|---|---|---|---|---|---|---|---|---|---|---|---|---|
| *Mouse grown tumours* | *cell yield*[a] $\times 10^7$ | *CFE*[b] % | *clon. yield*[c] $\times 10^6$ | *Nu*[d] | *cell yield* $\times 10^7$ | *CFE* % | *clon. yield* $\times 10^6$ | *Nu* | *cell yield* $\times 10^7$ | *CFE* % | *clon. yield* $\times 10^6$ | *Nu* |
| CPH SCCL 049[g] | 5.0 | 3.4 | 1.6 | + | 4.7 | 2.2 | 0.9 | + | 0.3 | 0.9 | 0.03 | + |
| CPH SCCL 054 B | 5.5 | 2.2 | 1.2 | + | 6.1 | 0.9 | 0.5 | + | 1.0 | 0.4 | 0.04 | + |
| CPH SCCL 075–5 | 5.5 | 3.0 | 1.6 | + | 0.6 | 0.03 | 0.02 | – | 0.03 | 1.2 | 0.03 | – |
| CPH SCCL 084 | 3.1 | 6.6 | 1.9 | + | 1.0 | 2.7 | 0.3 | + | <0.01 | <0.01 | <0.01 | [f] |
| T 60[h] | 2.4 | [e] | [e] | + | 0.1 | [e] | [e] | – | <0.01 | [e] | [e] | [f] |
| T 61 | 2.2 | [e] | [e] | + | 2.0 | [e] | [e] | – | <0.01 | [e] | [e] | [f] |
| T 2[i] | 10.0 | 0.9 | 0.7 | + | 3.0 | 1.0 | 0.2 | + | 0.1 | 0.01 | <0.01 | [f] |

[a]viable cell yield estimated by dye exclusion test
[b]colony forming efficiency
[c]clonogenic cell yield = number of colony forming cells $g^{-1}$ tissue
[d]ability to form tumours in nude mice
[e] not done since the breast carcinoma did not grow *in vitro*
[f] not done due to low yield of tumour cells
[g]small cell carcinoma of the lung
[h]breast carcinoma
[i]malignant melanoma

**Figure 1** Flow cytometric DNA distribution of breast carcinoma T60. The parts of the histograms produced by $G_1$, $S$, and $G_2+M$ cells are indicated in the figure. The peaks marked $D$ represent diploid mouse stromal cells, and the peaks $C$ and $T$ are internal standards used to calculate the DNA index. The DNA index is indicated in parentheses. The CV of $G_1$ peaks was 0.03. (a) The DNA distribution before disaggregation. $G_1=80\%$, $S=14\%$, $G_2+M=6\%$. Diploid cells = 8%, tumour cells = 92%. (b) The DNA distribution of the tumour after disaggregation by the long-term trypsinization procedure. $G_1=76\%$, $S=14\%$, $G_2+M=10\%$. Diploid cells = 8%, tumour cells = 92%. (c) The DNA distribution of the tumour after disaggregation by the short-term procedure. Diploid cells ≙ 92%, tumour cells = 8%. The distribution of cells in the cell cycle phases were not calculated.

**Figure 2** DNA distribution of CPH SCCL 094 obtained directly from the patient. The peaks $C$ and $T$ represent internal standards used to calculate the DNA index. The DNA index is indicated in parentheses. The parts of the histograms produced by $G_1$, $S$, and $G_2+M$ cells are indicated in the figures. (a) The DNA histogram of the tumour before disaggregation. (b) The DNA histogram of the tumour after disaggregation by the long-term trypsinization procedure.

**Table II** Disaggregation of human tumour tissue obtained direct from patients. The tumours were disaggregated by the long-term trypsinization procedure.

| *Fresh tumour specimens* | *yield* $\times 10^7$ [a] | *CFE* [b] |
|---|---|---|
| CPH SCCL 092[c] | 2.0 | 2.5 |
| CPH SCCL 094 | 5.0 | 0.1 |
| CPH TC 100[d] | 1.5 | 5.2 |
| CPH TC 102 | 5.0 | ND |

[a]Viable cell yield estimated by dye exclusion test
[b]Colony forming efficiency
[c]Small cell carcinoma of the lung
[d]Testicular cancer
ND not done

### Discussion

This study has shown that combined mechanical and enzymatic methods are appropriate for the disaggregation of a number of human solid tumours grown in nude mice or taken directly from patients. In contrast, the mechanical method was unsatisfactory with a view to cell yield and biological viability, i.e., the clonogenic yield and the tumorigenicity of the disaggregated cells.

The clonogenic cell yield of the long-term trypsinization technique was superior to that of the short-term method. Only long-term trypsinization resulted in a cell yield representative of all the tumours investigated as evaluated by flow cytometry and histology.

Since all the heterotransplanted tumours were aneuploid and murine stromal elements do not differ very much from human stromal cells (DNA index: 0.98), the fraction of normal cells in the supensions could be calculated. This ensures that the detected variations in plating efficiency after disaggregation by the different methods are real and not only reflect different amounts of stromal components in the suspensions.

The number of tumour cells $g^{-1}$ of tissue depends on the tumour. Generally, $5 \times 10^8 - 10^9$ cells have been calculated (Slocum *et al.*, 1980). Since heterotransplanted tumours are often very necrotic (Spang-Thomsen *et al.*, 1980), the yield of $\sim 5 \times 10^7$ viable cells obtained represents at least 10% of the total number of cells. This is considered satisfactory compared with the results of other methods for disaggregation of experimental tumours: $8 \times 10^7$ (Pretlow *et al.*, 1977), $1 \times 10^9$ (Reinhold, 1965) as well as human solid tumours: $2.4 \times 10^7$ (Hemstreet *et al.*, 1980), $4 \times 10^7$ (Rasey & Nelson, 1980), $5 \times 10^7$ (Eremin *et al.*, 1982), and $5 \times 10^6$ (Reinhold, 1965), especially because flow cytometric DNA analysis indicated that the yield was representative of the parent tumours after the long-term incubation procedure.

It is a common experience that it is difficult to grow breast cancer cells *in vitro* (Von Hoff *et al.*, 1981). In the present study, the mechanical and short-term disaggregation methods of the breast tumour T60 resulted in a cell yield, comprising stromal cells with only a very small fraction of tumour cells or none at all (Figure 1). Mechanical disaggregation methods are often used in the establishment of cell lines and our results indicate that one explanation for the difficulties in growing breast cancer could be the lack of tumour cells in the yield.

Long-term exposure to trypsin is cytotoxic for living cells *in vitro* (Grabstein & Cohen, 1965; Hodges *et al.*, 1973). However, the penetration of trypsin is temperature dependent and negligible at 4°C whereas the enzyme still has some effect on the cell surface at this temperature (Hodges *et al.*, 1973). This may explain why long-term trypsin incubation at 4°C results in a higher yield of clonogenic cells than short-term incubation at 37°C.

However, the use of enzymes in the disaggregation of tumours may result in changes in biological properties. The chemosensitivity of tumour cells may be dependent on the procedure applied (Rasey & Nelson, 1980), and trypsin is known to destroy membranes bound immunoglobulin (Russell *et al.*, 1976). Furthermore, the use of enzymes may affect important cell markers (Perussia *et al.*, 1979). Thus, it is important to be aware that representativeness determined by flow cytometric DNA analysis only, does not ensure representativeness for all other tumour parameters.

Fine-needle aspiration biopsy is a valid procedure by which to obtain representative cytological material from malignant tumours (Söderström, 1966). Furthermore, fine-needle aspiration samples for flow cytometric DNA analysis has been applied in a number of studies of human tumours in patients and in nude mice (Vindeløv, 1977, 1983*b*; Vindeløv *et al.*, 1982; Spang-Thomsen *et al.*, 1984; Rofstad *et al.*, 1982). Therefore, flow cytometric DNA analysis performed on tissue obtained by fine-needle aspirations was considered a valid baseline for comparison. The results showed that flow cytometry is an appropriate and rapid method for checking the representativeness of tumour cell suspensions. Furthermore, the method can measure the ratio of stromal cells to that of the tumour cells, if the DNA index of the tumour cells differs sufficiently from one. The results demonstrate that high cell yield and the estimation of the number of viable cells by the exclusion dye test are insufficient to ensure the representativeness of disaggregated tumour cells.

*This work was supported by grants from the Danish Cancer Society, the Danish Medical Research Council, and the Lundbeck Foundation.

### References

AGREZ, M.V., KUVACH, J.S. & LIEBER, M.M. (1982). Cell aggregates in soft agar "human tumour stem-cell assay". *Br. J. Cancer*, **46**, 880.

BRÜNNER, N. & VISFELDT, J. (1982). Histological changes following oestradiol treatment of a hormone-responsive human breast carcinoma grown in nude mice. *Acta Pathol. Microbiol. Immunol. Scand. Sect. A*, **90**, 355.

BRÜNNER, N., SPANG-THOMSEN, M., VINDELØV, L. & NIELSEN, A. (1983). Effect of $17\beta$-oestradiol on growth curves and flow cytometric DNA distribution of two human breast carcinomas grown in nude mice. *Br. J. Cancer*, **47**, 641.

CHRISTENSEN, I.J., HARTMANN, N.R., KEIDING, N., LARSEN, J.K., NOER, H. & VINDELØV, L. (1978). Statistical analysis of DNA distributions from cell populations with partial synchrony. In *Pulse Cytometry*, p. 71 (Ed. Lutz). European Press: Ghent.

COURTENAY, V.D., SELBY, P.J., SMITH, J.E., MILLS, J. & PECKHAM, M.J. (1978). Growth of human tumour cell colonies from biopsies using two soft-agar techniques. *Br. J. Cancer*, **38**, 77.

ENGELHOLM, S.A., SPANG-THOMSEN, M. & VINDELØV, L.L. (1983). A short-term in vitro test for tumour sensitivity to adriamycin based on flow cytometric DNA analysis. *Br. J. Cancer*, **47**, 497.

ENGELHOLM, S.A., BRÜNNER, N., SPANG-THOMSEN, M. & VINDELØV, L. (1984). Genetic instability of human solid tumours grown in nude mice. In *Immune-Deficient Animals*, p. 339 (Ed. Sordat). S. Karger: Basel.

EREMIN, O., COOMBS, R.A., PROSPERO, T.D. & PLUMB, D. (1982). T-lymphocyte and B-lymphocyte subpopulations infiltrating human mammary carcinomas. *J. Nat Cancer Inst.*, **69**, 1.

GRABSTEIN, G. & COHEN, J. (1965). Collagenase: Effect on the morphogenesis of embryonic salivary epithelium *in vitro*. *Science*, **150**, 626.

HAMBURGER, A.W. & SALMON, S.E. (1977). Primary bioassay of human tumor stem cells. *Science*, **197**, 461.

HEMSTREET, G.P., ENOCH, P.G. & PRETLOW, T.G. (1980). Tissue disaggregation of human renal cell carcinoma with further isopyknic and isokinetic gradient purification. *Cancer Res.*, **40**, 1043.

HODGES, G.M., LIVINGSTON, D.C. & FRANKS, L.M. (1973). The localization of trypsin in cultured mammalian cells. *J. Cell Sci.*, **12**, 887.

PAVELIC, Z.P., SLOCUM, H.K., RUSTUM, Y.M., CREAVEN, P.J., KARAKONSIS, C. & TAKITA, H. (1980). Colony growth in soft agar of human melanoma, sarcoma, and lung carcinoma cells disaggregated by mechanical and enzymatic methods. *Cancer Res.*, **40**, 2160.

PERUSSIA, B., TRINCHIERI, G. & CAROTTINI, J.-C. (1979). Functional studies of Fc receptor bearing human lymphocytes: effect of treatment with proteolytic enzymes. *J. Immunol.*, **123**, 681.

PRETLOW, T.P., GLOVER, G.L. & PRETLOW, T.G. (1977). Separation of lymphocytes and mast cells from the furth transplantable mast cell tumor in an isokinetic gradient of ficoll in tissue culture medium. *Cancer Res.*, **37**, 578.

RASEY, J.S. & NELSON, N.J. (1980). Response of an in vivo – in vitro tumour to X-rays and cytotoxic drugs: effect of tumour disaggregation methods on cell survival. *Br. J. Cancer*, **41**, suppl. IV, 217.

REINHOLD, H.S. (1965). A cell dispersion technique for use in quantitative transplantation studies with solid tumours. *Eur. J. Cancer*, **1**, 67.

ROFSTAD, E.K., FODSTAD, Ø. & LINDMO, T. (1982). Growth characteristics of human melanoma xenografts. *Cell Tissue Kinet.*, **15**, 545.

RUSSELL, S.W., DOE, W.F., HOSKINS, R.G. & COCHRANE, C.G. (1976). Inflammatory cells in solid murine neoplasms. I. Tumor disaggregation and identification of constituent inflammary cells., *Int. J. Cancer.*, **18**, 322.

SALMON, S.E., HAMBURGER, A.W., SOEHNLEN, B.S., DURIE, B.G.M., ALBERTS, D.S. & MOON, T.E. (1978). Quantitation of differential sensitivity of human-tumor stem cells to anticancer drugs. *N. Engl J. Med.*, **298**, 1321.

SLOCUM, H.K., PAVELIC, Z.P. & RUSTUM, Y.M. (1980). An enzymatic method for disaggregation of human solid tumors for studies of clonogeneity and biochemical determinants of drug action. In *Cloning of Human Tumor Stem Cells*, p. 339 (Ed. Salmon). Allan R. Liss: New York.

SPANG-THOMSEN, M., NIELSEN, A. & VISFELDT, J. (1980). Growth curves of three human malignant tumors transplanted to nude mice. *Exp. Cell Biol.*, **48**, 138.

SPANG-THOMSEN, M., BRÜNNER, N., ENGELHOLM, S.A. & VINDELØV, L. (1984). Estimation by flow cytometric DNA analysis of the effect of radiotherapy, hormone therapy and chemotherapy on human tumors grown in nude mice. In *Immune-Deficient Animals*, p. 409 (Ed. Sordat). S. Karger: Basel.

SÖDERSTRÖM, N. (1966). *Fine-Needle Aspiration Biopsy*. Grune & Stratton, New York.

VINDELØV, L.L. (1977). Flow microfluorometric analysis of nuclear DNA in cells from solid tumors and cell suspensions. *Virchows Arch* (*Cell Pathol.*), **24**, 227.

VINDELØV, L.L., HANSEN, H.H., GERSEL, A., HIRSCH, F.R. & NISSEN, N.I. (1982). Treatment of small cell carcinoma of the lung monitored by sequential flow cytometric DNA analysis. *Cancer Res.*, **42**, 2499.

VINDELØV, L.L., CHRISTENSEN, I.J., KEIDING, N., SPANG-THOMSEN, M. & NISSEN, N.I. (1983*a*). Long-term storage of samples for flow cytometric DNA analysis. *Cytometry*, **3**, 317.

VINDELØV, L.L., CHRISTENSEN, I.J. & NISSEN, N.I. (1983*b*). A detergent-trypsin method for the preparation of nuclei for flow cytometric DNA analysis. *Cytometry*, **3**, 323.

VINDELØV, L.L., CHRISTENSEN, I.J. & NISSEN, N.I. (1983*c*). Standardization of high-resolution flow cytometric DNA analysis by simultaneous use of chicken and trout red blood cells as internal reference standards. *Cytometry*, **3**, 328.

VON HOFF, D.D., SANDBACH, J., OSBORNE, C.K. & 6 others. (1981). Potential and problems with growth of breast cancer in a human tumor cloning system. In *Breast Cancer Research and Treatment 1*, p. 141. Martinus Nijhoff Publishers: Hague.

WILLSON, J.K., Jr., ZAREMBA, J.L., PITTS, A.M. & PRETLOW, T.G. (1976). A characterization of human tonsillar lymphocytes after separation from other tonsillar cells in isokinetic gradient of ficoll in tissue culture medium. *Am. J. Pathol.*, **83**, 341.